# Nematode Infections in Commercially Important Squid Species: Distribution Patterns and Food Safety Issues

**DOI:** 10.1111/zph.13221

**Published:** 2025-04-25

**Authors:** Monica Caffara, Perla Tedesco, Teresa Pirollo, Ahmed Abdelfadel, Riccardo Forzano, Maria Letizia Fioravanti, Andrea Gustinelli

**Affiliations:** ^1^ Department of Veterinary Medical Sciences (DIMEVET), Alma Mater Studiorum—University of Bologna Bologna Italy; ^2^ Fiorital SpA Venezia Italy

**Keywords:** *Anisakis* spp., cephalopods, *Hysterothylacium* spp., *I. coindetii*, *I. illecebrosus* and *Todaropsis eblanae*, *Illex* sp., *Lappetascaris* spp.

## Abstract

**Introduction:**

Cephalopods represent a valuable fishery resource worldwide and play a crucial role in the marine food chain both as predators and prey but also as paratenic hosts for the transmission of Ascaridoids, including zoonotic nematodes of the Anisakidae family. This study aimed to assess the presence, tissue distribution and species composition of zoonotic parasites in four squid species marketed in Italy, coming from four different FAO areas.

**Methods:**

A total of 238 squid specimens, *Illex* sp., *I. coindetii* , *I. illecebrosus* and *Todaropsis eblanae* , were caught across four FAO areas and examined using both visual inspection and the UV‐press method. The collected larvae were identified by morphological and molecular methods.

**Results:**

Third stage larvae of nematodes were detected in 22.3% of the squids (18.8% *Illex*sp., 18.3% *I. coindetii* , 48.8% *I. illecebrosus* and 23% *Todaropsis eblanae* ), with *Anisakis simplex* (*s.s*), *A. pegreffii*, *Hysterothylacium* spp. and *Lappetascaris* spp. being identified through morphological and molecular analyses. The UV‐press method has proved to be significantly more effective than visual inspection. Additionally, the lack of molecular data on Raphidascarididae species, particularly *Lappetascaris*spp., complicates taxonomic classification, emphasising the need for an integrative taxonomic approach combining morphological and genetic methods.

**Conclusion:**

Given the increasing consumption of raw and undercooked cephalopods, these findings highlight the importance of monitoring zoonotic parasites and improving molecular techniques to enhance food safety regulations and minimise health risks to consumers.

**Summary**

- Assess the presence and species composition of zoonotic Ascaridoid parasites in squid species marketed in Italy, highlighting the detection in the edible portion—the mantle—as relevant for food safety.
- The increased consumption of squid as sushi raises the need to assess the risk of acquiring zoonotic parasites.
- Establish official standardised diagnostic methods to generate new data for risk analysis.

## 1 Introduction

Cephalopods are increasingly recognised as a valuable fishery resource, with global catches and trade continuing to increase (FAO 2024). Particularly, ommastrephid squids (Oegopsida, Ommastrephidae) of the genera *Illex* and *Todaropsis* support some of the largest fisheries in the world.

The broadtail shortfin squid *Illex coindetii* is a demersal, neritic species of the continental shelf and upper slope; it is widely distributed in the Eastern Atlantic from the North Sea to Namibia and throughout the Mediterranean Sea, where it grows rapidly and reproduces throughout the year, with peak seasons varying according to geographic location. Its congeneric, the northern shortfin squid *Illex illecebrosus* , is also neritic, but distributed throughout the western Atlantic from Florida in the south to Labrador and Newfoundland waters in the north (Dawe and Beck 1985), and extends eastward to the British Isles. Both *Illex* species undergo diet shifts with growth, with smaller squids feeding mainly on crustaceans, while adult animals prefer preying on fishes and other cephalopods.

The lesser flying squid *Todaropsis eblanae* is a demersal species usually associated with sandy and muddy bottoms, and exhibits a very disjunct distribution (Roper et al. 2010). Similarly, *T. eblanae* shows an opportunistic feeding behaviour, exploiting a range of preys that include mainly fish and crustaceans, but occasionally even conspecifics.

From an ecological perspective, ommastrephid squids are a key link in the marine food chain both as predators and prey (Coll et al. 2013) and are therefore an important trophic bridge for the transmission of parasites in the marine ecosystem (Abollo et al. 1998). This is particularly true for nematodes of the family Anisakidae, with the squid species *I. coindetii* , *I. argentinus* , *T. eblanae* and *T. sagittatus* being the most important cephalopod paratenic hosts in the life cycle of *Anisakis* (Abollo et al. 1998; Gutiérrez et al. 2025), with prevalence values as high as 100% particularly among larger squids (González and Kroeck 2000), possibly because of ontogenetic diet shifts (Cipriani et al. 2019).

In squids, anisakid nematodes are found over the outer and inner membranes of the visceral mass, particularly over the gonads, where they have been hypothesized to cause parasitic castration in heavily infected individuals (Abollo et al. 1998). Other infection sites are the nidamental glands, the stomach wall and the mantle (Hochberg 1990; Palomba et al. 2021); in fresh dead squids, vital anisakid larvae can be detected inside the mantle cavity, posing inspection issues and determining the discard of the affected stock (Abollo et al. 2001).

The high prevalence and intensity of anisakids in commercially important squid species, together with the zoonotic aspect of certain anisakids, represent a serious food safety issue (Audicana et al. 2002; Mattiucci et al. 2013); therefore, the risk of exposure to potentially zoonotic nematodes through the consumption of squid products should be carefully assessed (Bao et al. 2017; Mattiucci et al. 2018; EFSA 2010, 2024) as evidenced by recent cases of human anisakiasis linked to the consumption of raw squid in Japan (Ogata et al. 2015; Tamai and Kobayashi 2015; Furuya et al. 2018). Recently, Cipriani et al. (2019, 2021) detected *A. pegreffii* in the mantle of *I. argentinus* and *A. simplex* (*s.s*.) in *T. sagittatus* ; albeit at a lower infection intensity, its presence still poses a direct risk to consumers, reinforcing the need for accurate parasite monitoring and risk assessment (Bao et al. 2017; Cipriani et al. 2021).

Furthermore, Anisakidae are increasingly recognised for their usefulness as biological tags for fishery stocks (Mattiucci et al. 2008; Timi and Buchmann 2023) and cephalopod stocks have been considered only recently as indicators of stock structure (Gutiérrez et al. 2023).

The aims of this preliminary study were to assess the presence, tissue distribution and species composition of zoonotic parasites in four squid species, namely *Illex coindetii* , *I. illecebrosus* , *Illex* sp. and *Todaropsis eblanae* , marketed in Italy, with particular attention to the occurrence of larvae in edible tissues and their implications for public health.

## 2 Materials and Methods

From November to December 2023, a total of 238 squids were caught during eight sampling events by bottom trawl method across four FAO areas. These squids belonged to the species *Illex* sp., *Illex coindetii, I. illecebrosus* and *Todaropsis eblanae* (Table 1). The squids were delivered to the Fish Pathology Unit (DIMEVET‐UNIBO) for visual inspection under good light conditions, with a focus on Anisakid nematodes in accordance with the ‘Hygiene Package’ 853/2004 and its amendments, as well as EU Regulation 2074/2005 and EU Directive 1276/2011.

**TABLE 1:** Details of the squid species sampled in November–December, together with the FAO fishing areas.

Species	N. squid	Mean weight (min − max ± SD)	FAO area
*Illex coindetii*	90	64.7 (13 − 291 ± 196.57)	Area 27 Atlantic Northeast Subarea 27.9A‐Portuguese water
*Illex* sp.	90	51.3 (13 − 128 ± 81.31)	Area 37 Mediterranean and Black Sea Subarea Gulf of Lyon (37.1.2)
*I. illecebrosus*	45	163.1 (72 − 314 ± 171.12)	Area 27 Atlantic Northeast Subarea 27.8‐Bay of Biscay
*Todaropsis eblanae*	13	107.2 (69 − 162 ± 65.76)	Area 27 Atlantic Northeast Subarea 27.8‐Bay of Biscay

Following visual inspection, the internal organs and mantle were individually placed in plastic bags and subjected to the UV‐press method (Gómez‐Morales et al. 2018; ISO 23036‐1:2021 Microbiology of the food chain—Methods for the detection of Anisakidae L3 larvae in fish and fishery products Part 1: UV‐press method). Specifically, the samples were pressed to a thickness of 2 mm and frozen at −20°C for at least 24 h before examination under 366 nm UV‐light in a dark room. Prevalence, mean intensity (MI) and mean abundance (MA) for each of the squid species and locality were calculated according to Bush et al. (1997).

The nematodes recovered after visual inspection were washed in saline and preserved in 70% EtOH for morphological and molecular identification, while the ones collected after UV‐press were stored at −20°C for molecular analysis. The DNA extraction was performed by the 5% Chelex100 method (Caffara et al. 2023) and the samples stored at −20°C until further use. DNA amplification was carried out on the ITS rDNA region with the primers NC5 and NC2 (Zhu et al. 1998). For the PCR‐RFLP, the PCR products were digested with the restriction enzymes *Hinf*I and *Hae*III (D'Amelio et al. 2000) at 37°C for 120 min (Abollo et al. 2003). The restriction fragments were separated in 2% agarose gel stained with SYBR Safe DNA Gel Stain in 0.5× TBE for 90 min. A representative sample of each restriction pattern was sent to the sequencing service (StarSEQ, Germany) after purification with NucleoSpin PCR & GEL Clean Up kit (Mackerey Nagel). The sequences obtained have been assembled and the identity verified by BLAST (https://blast.ncbi.nlm.nih.gov). The sequences from this study have been deposited in GenBank under the accession numbers PV244429-35.

The squid species examined during this study were provided by a food company intended for human consumption and comply with the Directive 2010/63/EU.

## 3 Results

**FIGURE 1:**
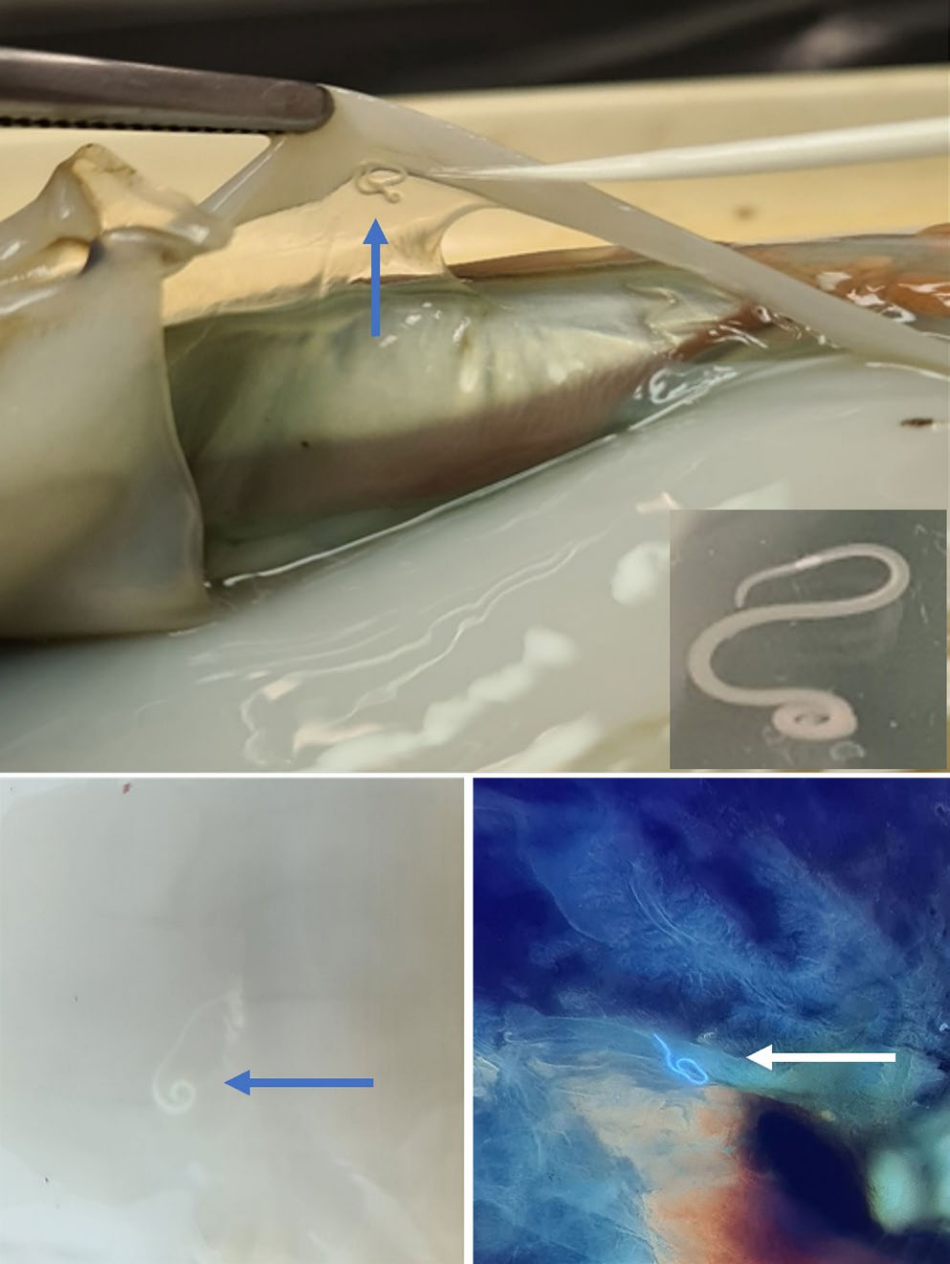
Detection of third‐stage larvae of Anisakidae and Raphidascarididae during visual inspection and UV‐press examination of mantle and viscera.

Of the 238 squids examined, 53 (22.3%) from the four species were positive for third‐stage larvae (L3) of nematodes, tentatively identified based on morphological features as belonging to the families Anisakidae and Raphidascarididae. The L3 were detected during visual inspection and UV‐press examination in both mantle and viscera (Figure 1). Overall, 45.3% of the L3 were found exclusively within the mantle, 39.6% exclusively in the viscera, while 15.1% were present in both tissues. During visual inspection, only 16 squids (6.7%) tested positive, whereas UV‐press examination detected nematodes in 37 squids (15.5%). Finally, in 6 squids (2.5%), nematodes were observed using both methods. Regarding the sampling seasons, prevalence values were similar in both months, 20.8% (25/120) in November and 23.7% (28/118) in December. As for the FAO fishing areas, all but the Aegean Sea tested positive, with prevalence values ranging from 18.3% to 43.1%.

Detailed results of the parasitological findings in the four squid species are reported in Table 2, along with a breakdown of prevalence (*P*) values, mean Abundance (mA) and mean Intensity (mI) in the different sites of infection (mantle and viscera) in the three FAO areas where positive squid were found.

**TABLE 2:** Detailed results of the parasitological findings in the four squid species along with a breakdown of prevalence (*P*), mean Abundance (mA) and mean Intensity (mI) values in the mantle, viscera and in mantle + viscera (total) in the three FAO areas.

Host	Ascaridoid	Mantle			Viscera			Total		
		*P*	mA	mI	*P*	mA	mI	*P*	mA	mI
*I. coindetii* (*n* = 90) Area 27 Atlantic Northeast Subarea 27.9A‐Portuguese water	*A. simplex* (*n* = 5)	2.2%	0.04	2	1.1%	0.01	1	3.3%	0.05	1.66
	*A pegreffii* (*n* = 4)	0%			2.2%	0.04	2	2.2%	0.04	2
	*Lappetascaris* sp. (*n* = 8).	3.3%	0.03	0.27	5.5%	0.05	0.45	6.6%	0.08	1.33
*Illex* sp. (*n* = 90) Area 37 Mediterranean and Black Sea Subarea Gulf of Lyon (37.1.2)	*Anisakis* sp. (*n* = 1)	0%			1.1%	0.02	1			
	*Lappetascaris* sp. (*n* = 14)	12.2%	0.12	0.78	3.3%	0.04	1.33	15.5%	0.16	1.07
	*Hysterothylacium* sp. (*n* = 2)	2.2%	0.02	1	0%					
*I. illecebrosus* (*n* = 45) Area 27 Atlantic Northeast Subarea 27.8C‐Bay of Biscay	*A. simplex* (*n* = 15)	6.6%	0.15	2.33	8.8%	0.17	2	13.3%	0.3	2.5
	*A pegreffii* (*n* = 9)	4.4%	0.04	1	4.4%	0.16	3.5	8.9%	0.2	2.25
	*Lappetascaris* sp. (*n* = 10)	6.6%	0.09	1.33	13.3%	0.13	1	20%	0.22	1.11
	Hybrid *A. simplex/A pegreffii* (*n* = 2)	0%			4.4%	0.04	1			
	*Hysterothylacium* sp. (*n* = 4)	2.2%	0.02	1	6.6%	0.06	3	2.2%	0.08	4
*T. eblanae* (*n* = 13) Area 27 Atlantic Northeast Subarea 27.8C‐Bay of Biscay	*A. simplex* (*n* = 2)	0%			15.4%	0.15	1			

*Note:* Host = squid species examined, number (*n*) of subject examined; Ascaridoid = Anisakids and Raphidascaridids species identified by molecular methods, number (*n*) of larvae collected.

**FIGURE 2:**
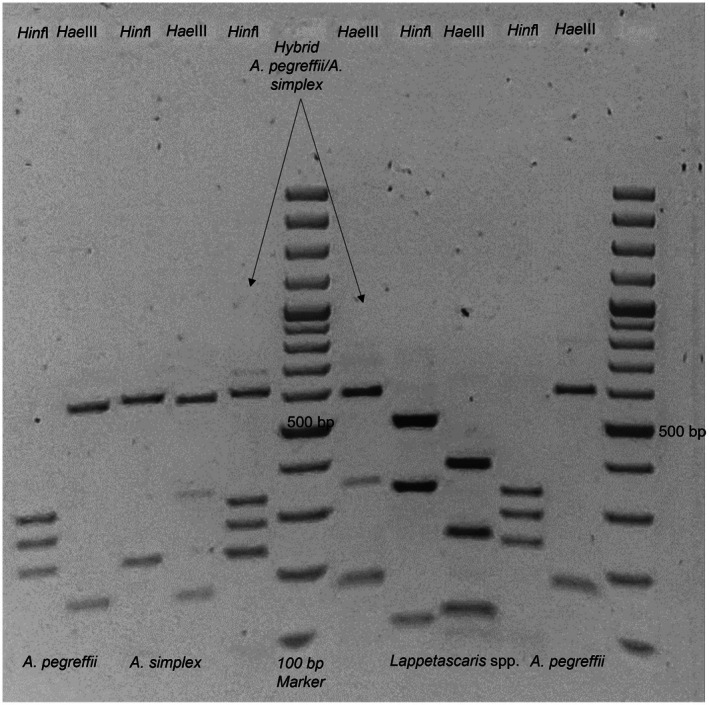
PCR‐RFLP patterns of *A. pegreffii*, *A. simplex*, *A. pegreffii*/*A. simplex* hybrid (arrows) and *Lappetascaris* spp. after digestion with the restriction enzymes *Hinf*I and *Hae*III.

A total of 84 L3 were collected and grossly identified as *Anisakis* spp. and *Hysterothylacium* spp. The PCR‐RFLP showed different restriction patterns that allow the identification among the Anisakidae of *Anisakis simplex* (*s.s*) and *A. pegreffii*, plus 2 hybrids *A. simplex/A. pegreffii*; while among the Raphidascarididae, *Lappetascaris* spp. and *Hysterothylacium* spp. The BLAST search of the representative sequences confirmed 100% identity for *A. simplex* and *A. pegreffii*. Concerning the Raphidascaridids, the results were unclear, showing 100% identity with both *Lappetascaris* sp. and *Hysterothylacium* sp. for the specimens with RFLP patterns that clearly separate the two genera (Figure 2).

The overall prevalence of *A. simplex* was 22.6%, in detail: *I. coindetii* (FAO 29.9A Portuguese water) 3.3% (mA 0.05, mI 1.66), *I. illecebrosus* (FAO 27.8C Bay of Biscay) 13.3% (mA 0.3, mI 2.5) and *T. eblanae* (FAO 27.8 Bay of Biscay) 2.2% (mA 0.08, mI 4). In 2 cases, we observed coinfections with *A. pegreffii* (1 *I. illecebrosus* ) and *Lappetascaris* sp. (1 *I. coindetii* ), respectively. *A. pegreffii* was recovered in 6 squids (11.3%), in detail: *I. coindetii* (FAO 29.9A Portuguese water) 2.2% (mA 0.04, mI 2) and *I. illecebrosus* (FAO 27.8C Bay of Biscay) 8.9% (mA 0.2, mI 2.25) and in 2 cases in coinfections with *Lappetascaris* sp. (2 *I. illecebrosus* ). The two hybrids *A. simplex/A. pegreffii* were detected in 2 *I. illecebrosus* from the Bay of Biscay fishing area (FAO 27.8C). The highest prevalence for *Anisakis* spp. (13.3% *A. simplex* (*s.s*.) and 8.9% *A. pegreffii*) has been observed in the largest squids (mean weight 198.5 g) belonging to *I. illecebrosus* species sampled in the Bay of Biscay. The most frequently recovered non‐zoonotic nematode was *Lappetascaris* sp. detected in 27 squids (11.3%): *Ilex* sp. (FAO 37.1.2 Gulf of Lion) 15.5% (mA 0.16, mI 1.07), *I. coindetii* (FAO 29.9A Portuguese water) 6.6% (mA 0.08, mI 1.33) and *I. illecebrosus* (FAO 27.8C Bay of Biscay) 20% (mA 0.22, mI 1.11); in 24 subjects (45.3%) as a single infection, while in 3 cases in coinfections with *A. simplex* (1 *I. coindetii* ) or *A. pegreffii* (2 *I. illecebrosus* ). Finally, *Hysterothylacium* sp. was found in four squids (7.5%): *Ilex* sp. (FAO 37.1.2 Gulf of Lion) 2.2% (mA 0.02, mI 1) and *I. illecebrosus* (FAO 27.8C Bay of Biscay) 2.2% (mA 0.08, mI 4) always as a single infection (see Table 2 for details).

## 4 Discussion

Overall, the results obtained in this preliminary study on four different squid species showed the presence of the anisakids *A. simplex* and *A. pegreffii*, both well‐known zoonotic species, also in edible squid tissues. In cephalopods, these parasites are generally encysted on visceral organs but can also migrate to the edible mantle part (Guardone et al. 2018; Mattiucci et al. 2013, 2018; Cipriani et al. 2019, 2021). Additionally, the study identified at genus level the Raphidascaridids *Hysterothylacium* sp. and *Lappetascaris* sp. which are generally considered non‐zoonotic (Guardone et al. 2020; Bao et al. 2022).

To date, squids are reported to host *A. pegreffii*, *A. simplex* , *Skrjabinisakis physeteris* (formerly *Anisakis physeteris*), *Contracaecum* sp. and *Pseudoterranova decipiens* (Tedesco et al. 2020); all but one (*S*. *physeteris*, whose zoonotic potential is yet to be confirmed) are considered pathogenic to humans. Additionally, various species of the Raphidascarididae family, formerly classified under Anisakidae, have been reported worldwide, including *Hysterothylacium reliquens*, *Lappetascaris* sp. and *Raphidascaris* sp. (Tedesco et al. 2020; Palomba et al. 2021; Bao et al. 2022).

Among the Mediterranean Sea, *A. pegreffii* has been reported infecting *I. coindetii* in the Adriatic (Petrić et al. 2011) and *T. sagittatus* in the Ionian (Costa et al. 2012) as well as along the North African coasts of the Mediterranean (Farjallah et al. 2008); *S*. *physeteris* in *T. sagittatus* in the Tyrrhenian (Mattiucci et al. 2001) and in the Ionian (Costa et al. 2012); *A. simplex* in *I. coindetii* in the Tyrrhenian (Gestal et al. 1999) and *T. sagittatus* from France (Hochberg 1990). Reports from the Northeastern Atlantic include: *A. pegreffii* in *I. coindetii* (Picó‐Duran et al. 2016); *S. physeteris* in *Illex argentinus* (Picó‐Duran et al. 2016) and in *Ommastrephes bartramii* ; *A. simplex* in *I. coindetii* , *T. sagittatus* and *Todaropsis eblanae* (Nagasawa and Moravec 1995; Pascual et al. 1995, 1996, 1999; Abollo et al. 1998, 2001; González et al. 2003; Cipriani et al. 2021).

Concerning Raphidascaridids, most reports of *Hysterothylacium* and *Lappetascaris* in squids lack specific identification (Tedesco et al. 2020) being based solely on morphological analysis (Culurgioni et al. 2010; Guardone et al. 2020; Palomba et al. 2021; Bao et al. 2022). For these reasons, the application of an integrative taxonomic approach is essential to understanding the diversity and distribution of Raphidascaridids in cephalopods, as recently used by Cipriani et al. (2019) to identify *H. aduncum* in *I. argentinus* from the southwest Atlantic Ocean.

To date, the genus *Lappetascaris* comprises three species, all found in marine/brackish waters: *L. chandipurensis*, *L. lutjani* and *L. suraiyae*. Unfortunately, no molecular data of any of these species are so far available, which hinders our understanding of their epidemiology in fish and cephalopod, as reported also by other authors (Palomba et al. 2021; Bao et al. 2022). In squid, larvae of *Lappetascaris* sp. are grouped in two morphologically similar larval types differing mainly in body size, infection site and squid species: type A larva infects mainly the mantle musculature, while type B is found encapsulated in the stomach wall (Nagasawa and Moravec 1995, 2002; Culurgioni et al. 2010; Bao et al. 2022). Palomba et al. (2021) reported that most research carried out on cephalopods is based on morphological features of the L3, which do not allow for accurate taxonomic identification of the recovered larvae. The L3 stage lacks important morphological features necessary for species identification, and in some cases, even for genus‐level identification, such as in the case of *Lappetascaris*/*Hysterothylacium*. Moreover, neither the molecular analysis based on a single marker is useful to clearly separate the two genera (Palomba et al. 2021). Even though the Raphidascaridids collected in this study are not considered a zoonotic risk, their presence in the mantle could reduce the marketability of the squid, especially if consumed raw.

Recent review on parasite diversity in cephalopods (Tedesco et al. 2020) highlighted the scarcity of specific taxonomic information, particularly for Anisakid and Raphidascaridid infection reports; the increasing application of molecular methods for species identification has provided valuable insights in the life cycle and ecology of several anisakids, particularly underlining the role of squids in the life cycle of *S*. *physeteris* (Mattiucci et al. 2018). The squid species examined in the present study are often consumed raw as sushi or undercooked; therefore, the public health risk should be taken into consideration (Cipriani et al. 2021). Nevertheless, the risk associated with the viscera could be reduced through rapid evisceration, which also prevents the migration of the larvae into the mantle edible part.

Previous studies (Guardone et al. 2020; Menconi et al. 2020; Cipriani et al. 2019, 2021) have reported the presence of L3 stage either in the squid mantle or viscera, or both; Palomba et al. (2021) observed the presence of nematode larvae in both tissues of the same subject, consistent with our findings. However, the study of Cipriani et al. (2021) reported the presence of *A. simplex* (*s.s*.) larvae in the mantle portions of *T. sagittatus* , specifically in areas in close contact with infected viscera. They speculated that the larvae could penetrate the mantle *intra vitam*, suggesting a potential migration pathway occurring during the host's life. Currently, only visual inspection under natural light conditions and, if necessary, candling (trans‐illumination) is included in the EU food hygiene and safety regulation (EC n. 2074/2005) nevertheless, the international scientific community applies the UV‐press method. Our results confirmed that the observation of the specimens under UV light is the most sensitive method compared to visual inspection, as already reported for fish (Llarena‐Reino et al. 2012, 2013; Gómez‐Morales et al. 2018). The UV‐press method is not commonly used for cephalopods, and only a few studies used this method, such as Guardone et al. (2020) and Cipriani et al. (2019, 2021). However, in the fish industry, visual inspection is the only feasible method due to time and market value constraints.

The results obtained in the present preliminary research enrich the number of studies on the presence of zoonotic Anisakids in cephalopods marketed in Italy as requested by the European Food Safety Authority, that recommends investigation on the prevalence, intensity and anatomical location of parasites of public health importance in wild caught fishery products (EFSA 2010, 2024). Moreover, there is also a need to establish official diagnostic methods, although the visual inspection proved to be a less sensitive technique. Standardisation is therefore crucial to generate new data for risk analysis, enabling more effective implementation of inspection procedures by Food Business Operators, ensuring product quality and consumer safety.

## Conflicts of Interest

The authors declare no conflicts of interest.

## Data Availability

The data that support the findings of this study are available from the corresponding author upon reasonable request.
